# Workloads of Different Soccer-Specific Drills in Professional Players

**DOI:** 10.2478/hukin-2022-000075

**Published:** 2022-11-08

**Authors:** Marcos Chena, José Alfonso Morcillo-Losa, María Luisa Rodríguez-Hernández, Iván Asín-Izquierdo, Beatriz Pastora-Linares, Juan Carlos Zapardiel

**Affiliations:** 1 Department of Biomedical Sciences, Faculty of Medicine and Health Sciences, University of Alcalá, Madrid, Spain.; 2 Professional Soccer Conditioning Coach. Jaén, Spain; 3 Department of Didactic of Musical, Plastic and Corporal Expression, University of Jaén, Jaén, Spain.; 4 Faculty of Sport Science, European University of Madrid, Madrid, Spain.; 5 Physical Performance and Sports Research Center, Department of Sports and Computer Sciences, Faculty of Sport Sciences, Pablo de Olavide University, Seville, Spain.

**Keywords:** soccer, GPS, soccer-specific drills, training load

## Abstract

Soccer is a predominantly tactical sport and, therefore, tactical training has become the most widely used strategy to improve players' performance. The objective of the present study was to assess the workload of soccer-specific drills in professional players over a two-season period in an established context. GPS technology was used to record the data. One hundred and thirty-two (n = 132) soccer-specific drills were studied and grouped by categories. The individual demands of each task were related to the individual competitive profile of each player. The level of physical demand was significantly different in relation to the specific soccer drills analysed. Total distance covered, high-speed running, and the total number of high accelerations and decelerations were significantly higher in competition than in drills used for training sessions (p < .001). The Large-Sided Games (LSG), Big-Position Games (BPG) and Position Games (PG) showed higher maximum running speed values than the rest of the exercises (p < .01). The sum of high accelerations and decelerations values was greater in the Small-Sided Games (SSG) than in BPG (p < .001), Small-Position Games (SPG) (p < .001) and Physical-Technical Circuits (PTC) (p < .001). Significant differences were observed in the exercises analysed according to the player’s position. The current findings provide a detailed description of conditional demands placed on soccer players in different soccer-specific drills during training sessions, in a professional soccer context and according to their playing position, which may be helpful in the development of individualized training programs in other contexts.

## Introduction

The development of soccer in recent years has revealed the need to investigate variables related to the planning and designing of training (Barnes et al., 2014; Malone et al., 2015). Recent research shows that physical, technical and tactical abilities of soccer players have been compromised by a greater multifactorial requirement, with today’s soccer being faster, more intense and competitive than ever before (Barnes et al., 2014; Bush et al., 2015).

There is some evidence on the need to control certain technical and physical indicators in isolation in order to predict team performance (Bradley et al., 2009, 2010; Di Salvo et al., 2007, 2009; Głowacki et al., 2011; Mohr et al., 2005). However, it has been shown that success in a soccer team is due to multivariate and complex factors, where both physical and technical requirements are influenced by situational, contextual and tactical variables (Ade et al., 2016; Bradley and Ade, 2018; Bush et al., 2015; Morgans et al., 2018).

On the other hand, there is also a need to find an appropriate balance between stress caused by training-competition and recovery processes to ensure maximum competitive performance with the minimum risk of injury (Bowen et al., 2017; Malone et al., 2017; Owen et al., 2017). To this effect, the use of global positioning systems (GPSs) has become more and more frequent with the aim of controlling the training load (TL) in professional soccer players. These GPS units are reliable and precise enough to quantify the conditional requirements of soccer (Ehrmann et al., 2016; Owen et al., 2017).

Consequently, the analysis of team sports performance requires a multidimensional approach, in accordance with its own nature, that helps analyse the adaptive behaviour of players and teams. Soccer is a predominantly tactical sport (Ade et al., 2016; Bradley and Ade, 2018) and the execution of actions related to information processing and perceptual-cognitive skills are particularly important when the objective is to resolve complex conflicts generated by the confrontation between two teams (Lex et al., 2015; Vestberg et al., 2012; Williams et al., 2012). At present, specific training has become the most utilized strategy by coaches to improve performance of athletes (Sangnier et al., 2019). Soccer-specific drills can maximize the most relevant adaptations (Casamichana and Castellano, 2010; Casamichana et al., 2012; Impellizzeri et al., 2004), while minimizing the cumulative effects of fatigue (Hill-Haas et al., 2010), monotony and training stress (Issurin, 2010). The "knowledge structures" created as a result of practical experience favour the effectiveness in the processing of contextual information (Williams et al., 2012).

Modifications of the variables of space, game rules, and players’ density have been widely used in soccer match simulations in recent years to achieve specific adaptations of soccer players (Asian-Clemente et al., 2021; Casamichana and Castellano, 2010; Köklü et al., 2020; Sangnier et al., 2019). Coaches have designed many training drills to improve the tactical behaviours of soccer players, approaching the conditional demands of the match. In team sports settings, small-sided games (SSG) have been used as key context tools to stress the players' awareness of in-game required behaviours (Gonçalves et al., 2016). However, planning training based on tactical demands requires the control of physiological response of players undergoing this type of training.

Modifications of playing space, game rules, and the number of players have been widely used in soccer match simulations in recent years to achieve adaptations of player-specific conditional capabilities (Asian-Clemente et al., 2021; Casamichana and Castellano, 2010; Köklü et al., 2020; Sangnier et al., 2019). In this sense, coaches have designed training drills to improve the tactical behaviours of players and, at the same time, have tried to make these exercises as similar as possible to the conditional requirements of soccer matches. In the area of team sports, small-sided games (SSG) have been used as a training tool to improve players’ tactical behaviours based on the needs of the game (Gonçalves et al., 2016). However, in order to plan training including SSG, it is necessary to establish not only the tactical demands of these games, but also the physical demands when performing this type of training.

Therefore, a responsibility of fitness coaches and sport scientists is to control the physical-physiological demands of different drills designed throughout the competitive season in order to schedule them in the competition calendar, attending to the contextual needs of each team. The aim of the present study was to monitor the workload of soccer-specific drills used in training sessions during two full seasons, in a professional team context, with 43 soccer players according to their playing position.

## Methods

### Participants

Forty-three Spanish professional soccer players participated in the study. The average age, body height, body mass, body fat content, VO_2max_ (Yo-Yo Intermittent Recovery Test level 2) and the sum of 6 skinfolds were: 26.13 ± 3.2 years, 178 ± 4.3 cm, 73.87 ± 7.6 kg, 9.68 ± 6.4%, 53.21 ± 4.7 ml·kg^-1^ and 39.58 ± 11.4 mm, respectively. Signed informed consent was received from all players after a detailed explanation about the research was given. Players were free to withdraw their consent and information from the study at any time. The study was fully approved by the involved soccer club. Furthermore, this study was approved by the ethics committee of the research university (CEI / HU / 2019/08) and was carried out in accordance with the ethical standards of the Declaration of Helsinki.

### Measures

Variables recorded from GPS devices for the purpose of this study were: maximum speed reached (maximum speed), total distance covered (TDC), high-speed running (HSR) and the sum of high intensity efforts, which is the total number of high accelerations and decelerations (Ac:Dc). All variables recorded, except maximum speed (km/h), were reported in both absolute (m) and relative terms (m/min) (Table 1). The specific speed thresholds set were in line with previous research (Casamichana et al., 2012).

**Table 1:** Variables’ definition.

Absolute variables	
Total distance covered (TDC) (m)	Total distance covered in meters
High-speed running (HSR) (m)	Total distance covered > 21 km/h
Total number of high accelerations and decelerations (Ac:Dc)	Sum of high intensity efforts which is the total number of high accelerations and decelerations (Sum Ac:Dc > 2.5 m/s^2^)
**Relative variables**	
Relative distance covered (RTDC) (m/min)	Total distance covered in meters per minute
Relative high-speed running (RHSR) (m/min)	Total distance covered > 21 km/h in meters per minute
Sum accelerations and decelerations relative to time of (RAc:high Dc) (nº/min)	Sum of high accelerations and decelerations relative to time
Top Speed	Maximum speed reached (km/h)

Each soccer player was analysed individually. The competitive profile was calculated as the mean value of the registered data from all the competition matches where the player participated for more than 80 min (Malone et al., 2015). Despite using various formations (game systems) in professional soccer teams during the seasons, players were grouped according to a 1-44-2 formation because it was the most common structure during matches and because all players could respond to the characteristics required in these positions (Bradley et al., 2009; Bradley and Ade, 2018; Di Salvo et al., 2009; Mohr et al., 2005). Data were grouped according to the following specific positions of soccer player: Central Defender (CD) (n = 9), Fullback (FB, n = 9), Central Midfielder (CM, n = 8), Wide Midfielder (WM, n = 10), Central Forward (CF, n = 7).

### Design and procedures

This was an observational research study conducted with 43 Spanish professional soccer players in a professional context during two full seasons. A total of 68 competitive matches and 132 different soccer-specific drills were recorded (those exercises in which the ball was not used were eliminated from the study). To ensure the reliability and validity of the study, the recorded data corresponded to players who successfully completed the drill, removing the data from goalkeepers and players who did not complete the drill due to fatigue or injury. All the participants had a least 6 weeks' experience using GPS devices. Preseason values were not considered.

In addition to the Official Game (Match Game), all specific soccer exercises were grouped according to their specific characteristics of structure (Casamichana and Castellano, 2010) and specific qualities, their approaching levels or nature considering previously published methodologies used in professional Spanish soccer (Pons et al., 2020): Large-Sided Games (LSG), Big Position Games (BPG), Small Position Games (SPG), Small-Sided Games (SSG), Possession Games (PG), and Physical-Technical Circuits (TC). In Table 2, the description of each category of exercises is provided. Strategy exercises and rondos (Pons et al., 2020) were not included in the analysis because they did not have a considerable load value.

**Table 2:** Soccer-specific drills taxonomy.

Levels of technical- tactical approach	Drills	Characteristics of each soccer-specific drill	Illustrative example
Minimum specificity level	Physical- Technical Circuits	Automated sequence of passes with or without a shot on the goal. These circuits can combine different displacement actions using various materials: rings, exercises ladders, hurdles, poles, cones, etc.	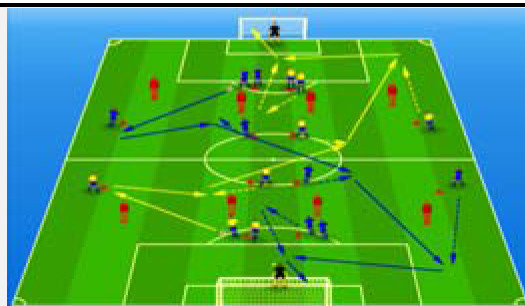 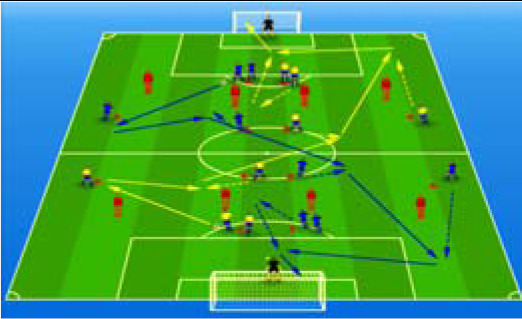
Low specificity level	Possession Games	Dynamic technical-tactical drill to keep the ball possession in a large or medium space (175-220 m^2^ per player) without specific playing positions and without a goalkeeper. In these exercises there may or may not be the presence of extra players to create numerical superiority.	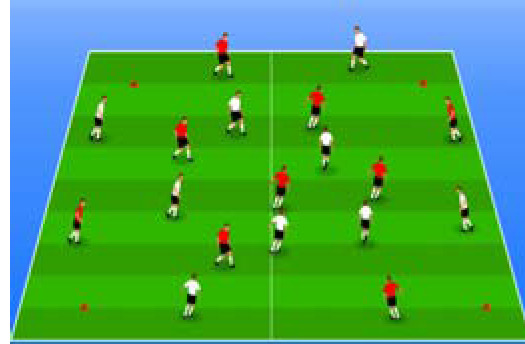
of specificity Low-moderate level	Games Small Position	Dynamic technical-tactical drill to keep playing position. In these exercises there the ball possession in a small space with defined specific directionality and a may or may not be the presence of extra players to create numerical superiority.	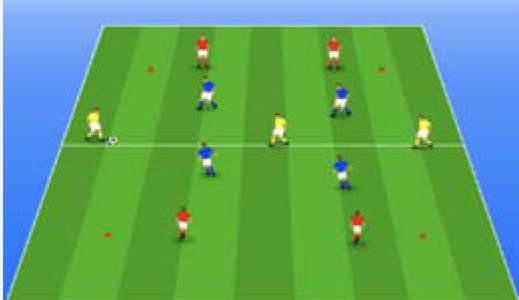
specificity Moderate level of	Games Big Position	playing position. In these exercises there Dynamic technical-tactical drill to keep the ball possession in a large space with defined specific directionality and a may or may not be the presence of extra players to create numerical superiority.	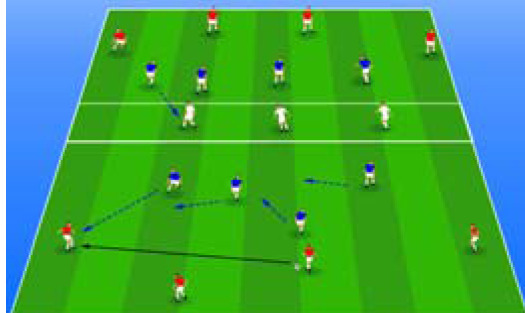
Moderate-high level of specificity	Small-Sided Games	Soccer matches played on smaller fields (75-200 m^2^ per player) with fewer players (3-5 field players plus a goalkeeper). In these exercises there may or may not be the presence of extra players to create numerical superiority.	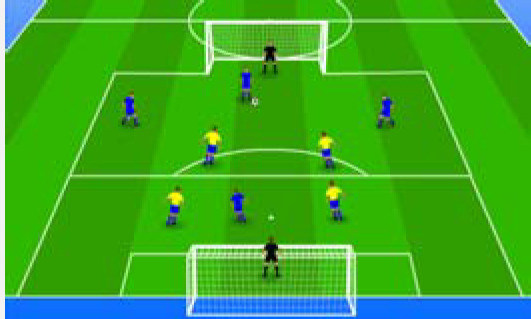
High specificity level	Large-Sided Games	Large-sided matches with official game rules and other added conditions (area ¾ of the pitch, box to box, box to the goal line) with less than 11 players per team (7-9 field players plus a goalkeeper). In these exercises there may or may not be the presence of extra players to create numerical superiority.	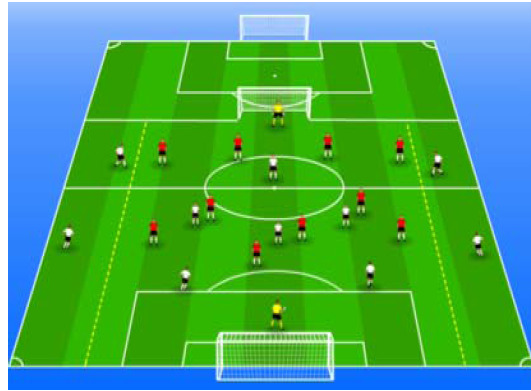
Maximum level specificity	Match Games	Official matches	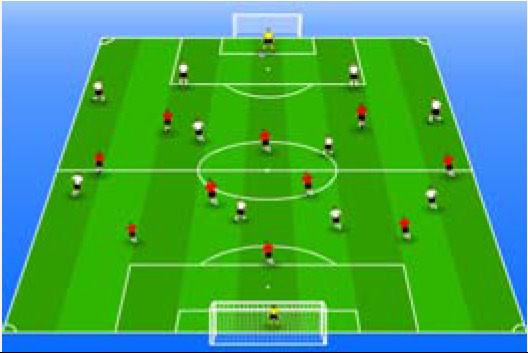

The time-motion analysis of each player was individually recorded in all training sessions and competition matches using a 10 Hz GPS device (GPEXE©. ExelioSRL, Udine, Italia), which has proven to be a reliable tool for monitoring performance variables (di Prampero et al., 2015). GPSs have been described as a reliable tool to determine external loads in team sports (di Prampero et al., 2015), and there are high correlations with other variables related to the workload, such as s-RPE (Ehrmann et al., 2016). The devices were switched on 15 min before each session according to the manufacturer’s regulations. To avoid measurement variability, each player always used the same GPS device located between the two scapulae using a special vest.

### Statistical analysis

The descriptive statistics of this study are presented as mean values and standard deviations (± SD). Before applying parametric tests, the normality of the data was verified by the Shapiro-Wilk test. A one-way analysis of variance with repeated measures (ANOVA) was used to test the differences in performance variables in each soccer-specific drill. In the event of a significant difference, Tukey's post-hoc tests were used to identify any localized effects. The effect sizes for these differences were also determined. Effect size values of .20-.49; .50-.79; and ≥ .8 represented small, medium, and large differences, respectively (Cohen, 1988).

Analyses were performed using SPSS for Mac version 24.0 (SPSS Inc., Chicago, IL, USA). The level of significance was set at *p* < .05.

## Results

A total of 68 competitive matches and 132 different soccer-specific drills were recorded (Physical-Technical Circuits, n = 19; Position Games, n = 20; Small-Position Games, n = 23; Big-Position Games, n = 26; Small-Sided Games, n = 25; Large-Sided Games, n = 19).

**Figure 1:**
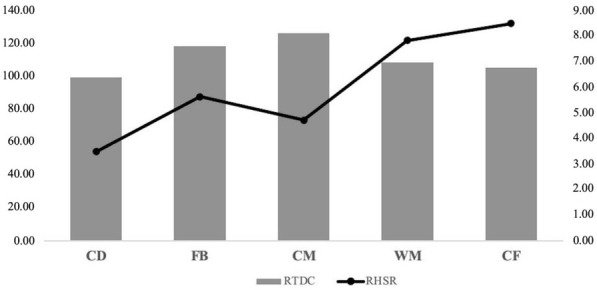
Competitive physical performance profile according to the playing position. Average data of relative distance covered (RTDC) and relative high-speed running (RHSR). CD = Central Defender; FB = Fullback; CM = Central Midfielder; WM = Wide Midfielder; CF = Central Forward.

Figure 1 shows the competitive physical performance profile according to the relative variables value. Competitive physical performance of soccer players was different depending on their playing position. Referencing these relative values, it was observed that Central Forwards and Wide Midfielders showed the highest Relative High-Speed Running variable values, and Central Midfielders and Fullbacks showed the highest Relative Distance Covered variable values, although significant differences were not found.

**Figure 2:**
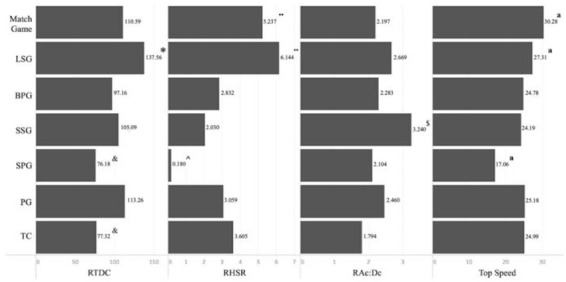
Relative distance covered (RTDC), relative high-speed running (RHSR), sum of high accelerations and decelerations relative to playing time **(**RAc:Dc) and Top speed in soccer-specific drills. ^*^Levels of significance found for RTDC (p < .01); ^&^Levels of significance found for RTDC from Match Game, LSG, BPG, SSG and PG (p < .01); ¨Levels of significance found for RHSR from BPG, SSG, SPG, PG and TC (p < .01); ^Levels of significance found for RHSR from Match Game, LSG, BPG, PG and TC (p < .05); ^$^Levels of significance found for RAc:Dc (p < .01); ªLevels of significance found for Top Speed (p < .01). LSG = Large-Sided Games; BPG = Big Position Games; SPG = Small Position Games; SSG = Small-Sided Games; PG = Possession Games; TC = Physical-Technical Circuits.

The level of the workload was significantly different depending on the specific soccer drills. Total Distance Covered, High-Speed Running and Ac:Dc were significantly higher in competition (Match Game) than in drills used for training sessions (*p* < .001). Greater distances were covered in large-space drills. LSG showed higher Relative Distance Covered values than the rest of the drills (*p* < .01), while SPG and Physical-Technical Circuits showed the lowest Relative Distance Covered values. Relative High-Speed Running was significantly higher in Match Game and LSG than in SSG (*p* < .001, d = 1.31), BPG (*p* < .001, d = 1.02) and SPG (*p* < .001, d = 1.91). However, RAc:Dc values were greater in SSG when compared with BPG (*p* < .001, d = 0.91), SPG (*p* < .001, d = 0.98) and Physical-Technical Circuits (*p* < .001, d = 1.60). Regarding maximum speed, Match Game and LSG showed the greatest values (*p* < .001), whereas SPG showed the lowest values (*p* < .001) (Figure 2).

Regardless of the playing position, all players showed greater values of total distance covered and running at high speed in LSG when compared with SPG (*p* < .05). Regarding exercises in a limited space, Fullbacks showed significantly more Ac:Dc in SSG when compared with SPG (*p* < .05, d = 2.14).

Midfielders and Central Forwards presented more Relative Distance Coverage in LSG (*p* < .01, d = 1.60; *p* < .05, d = 1.48), in BPG (*p* < .01, d = 4.57; *p* < .05, d = 5.25) and in PG (*p* < .001, d = 6.97; *p* < .01, d = 5.91) compared to SPG. The fullback, midfield, and central forward players showed higher relative high-speed runs in LSG when compared to SPG (*p* < .001, d = 2.56; *p* < .01, d = 1.85; *p* < .001, d = 2.29) and SSG (*p* < .05, d = 1.37; *p* < .05, d = 1.51; *p* < .05, d = 1.66). Central Midfielders showed higher Relative High-Speed Running in PG when compared with SSG (*p* < .05; d = 1.91) and lower when compared with LSG (*p* < .05; d = 1.27). SPG was the type of the drill where soccer players reached the lowest maximum speed, regardless of the playing position (*p* < .01) (Table 3).

**Table 3:** Soccer-specific drills average data according to the positional role.

		Absolute values			Relative values			
		TDC	HSR	Ac:Dc	RTDC	RHSR	RAc:Dc	Top speed
Match	Team	9981.36*	531.33*	197.30*	110.36*^#^*	5.90	2.19	30.21*^#$&+^^*
Game	CD	8710.00*	305.85*	151.00*	99.34	3.48	1.68	28.50*^#^*
	FB	10625.70*	506.40*	172.00*	118.06	5.62	1.91	31.30*^#+^^*
	CM	11371.85*	423.95*	179.50*	126.35*^#^*	4.71	1.99	27.85*^#^*
	WM	9748.20*	681.05*	243.50*	108.31	7.83	2.71	31.85*^#$&+^^*
	CF	9451.05*	759.40*	240.50*	105.12*^#^*	8.49*^#$^*^+^	2.67	31.56*^#$&+^^*
LSG	Team	2573.87	115.21	48.93	139.51	6.10	2.68	27.26
	CD	2287.45*^#^*	69.02*^#^*	39.85	124.01	3.55*^#^*	2.13	25.80*^#^*
	FB	2458.06*^#^*	137.90*^#$^*	48.92	132.77	7.45*^#$^^*	2.68	28.03*^#$^*
	CM	2834.61*^#^*^&^	79.81*^#^*	47.26	153.26*^#^*	4.07*^#^*	2.58	26.25*^#^*
	WM	2680.96*^#^*	174.29*^#^*	57.69	145.50	9.16*^#$^*	3.14	28.23*^#$^*
	CF	2397.48*^#^*	121.19*^#^*	50.46	130.33*^#^*	6.54*^#$^*	2.80	28.29*^#^*
BPG	Team	1497.56	42.50	34.01	99.24	2.81	2.25	24.68
	CD	1386.19	36.10	32.30	92.03	2.39	2.14	24.55*^#^*
	FB	1358.44	53.56	40.30	90.17	3.56	2.67	25.56*^#^*
	CM	1678.47	31.47	31.36	111.19*^#^*	2.09	2.08	23.65*^#^*
	WM	1458.17	47.78	34.87	96.53	3.15	2.31	25.52*^#^*
	CF	1413.27	45.40	33.97	93.81*^#^*	2.98	2.25	24.70*^#^*
SPG	Team	812.87	2.27	22.45	76.71	0.25	2.13	17.16
	CD	829.35	2.76	21.89	77.89	0.31	2.08	16.97
	FB	815.18	0.60	23.08	77.41	0.07	2.26	16.89
	CM	764.24	1.30	18.20	72.62	0.14	1.78	16.14
	WM	839.73	1.12	27.06	78.73	0.12	2.48	18.03
	CF	779.79	1.67	20.00	73.73	0.19	1.88	17.20
SSG	Team	1642.10	31.23	48.41	106.21	2.04	3.19	24.31
	CD	1532.09	18.44	40.42	99.43	1.31	2.66	23.47*^#^*
	FB	1672.47	52.45	56.40*^#^*	107.60	3.14	3.73	24.41*^#^*
	CM	1702.26	28.90	47.19	110.16	1.85	3.08	24.12*^#^*
	WM	1679.15	29.96	53.20	107.83	1.82	3.49	23.99*^#^*
	CF	1527.09	30.69	49.20	99.31	2.02	3.29	24.84*^#^*
PG	Team	1592.06	44.32	33.75	116.09	3.28	2.47	25.26
	CD	1427.51	30.96	31.42	103.93*^#^*	2.33	2.29	24.81*^#^*
	FB	1483.43	41.24	36.50	107.65*^#^*	3.12	2.68	25.12*^#^*
	CM	1790.29	41.49	31.76	130.74*^#&^*	2.98*^$^*	2.32	24.66*^#^*
	WM	1580.21	44.62	35.95	115.13*^#^*	3.35	2.63	25.49*^#^*
	CF	1453.53	44.18	32.29	106.03*^#^*	3.30	2.36	25.71*^#^*
TC	Team	894.13	40.50	19.62	77.61	3.56	1.75	24.82
	CD	917.44	43.41	20.50	79.55	3.75	1.83	24.02*^#^*
	FB	878.08	47.37	23.33	76.20	4.19	2.10	26.51*^#^*
	CM	885.12	38.95	17.70	76.96	3.40	1.58	25.04*^#^*
	WM	880.85	42.66	18.50	76.47	3.78	1.65	24.78*^#^*
	CF	889.67	33.26	20.69	77.15	2.96	1.84	24.77*^#^*

* Significant differences between all positions; ^#^significant differences with SPG; ^&^significant differences with TC; ^$^significant differences with SSG; ^^^significant differences with PG; ^+^significant differences with BPG. LSG = Large-Sided Games; BPG = Big Position Games; SPG = Small Position Games; SSG = Small-Sided Games; PG = Possession Games; TC = Physical-Technical Circuits; CD = Central Defender; FB = Fullback; CM = Central Midfielder; WM = Wide Midfielder; CF = Central Forward.

**Figure 3:**
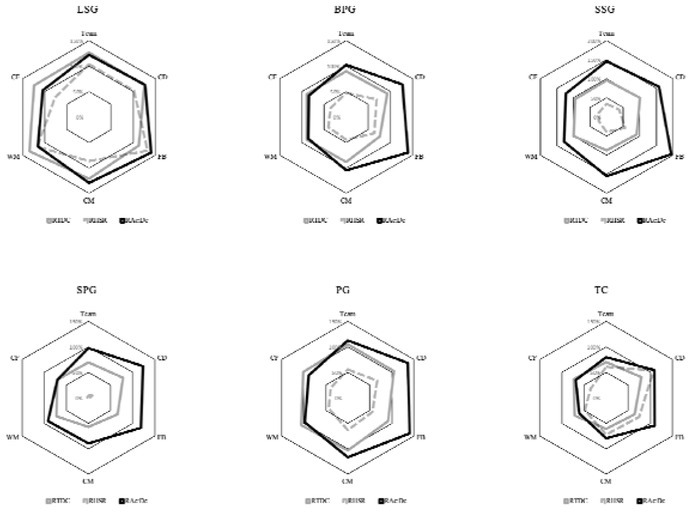
Relative distance covered (RTDC), relative high-speed running (RHSR) and sum of high accelerations and decelerations relative to playing time **(**RAc:Dc) in soccer-specific drills according to the playing position. Values expressed as % according to the competitive profile. LSG = Large-Sided Games; BPG = Big Position Games; SPG = Small Position Games; SSG = Small-Sided Games; PG = Possession Games; TC = Physical-Technical Circuits; CD = Central Defender; FB = Fullback; CM = Central Midfielder; WM = Wide Midfielder; CF = Central Forward.

Figure 3 shows the Relative Distance Covered, Relative High-Speed Running and RAc:Dc in soccer-specific drills according to the playing position. These results are expressed in % according to the competitive profile. According to these results, it was observed that relative load values were higher than in competition in several playing positions. All players showed RAc:Dc values higher than official games in SSG. However, FB players showed values close to 200% compared to the competitive profile.

## Discussion

This is one of the few studies in which the workload in soccer players in specific training drills performed during two full seasons has been described in a professional team context. To date, most of the studies published in this line of research had analysed soccer tasks classifying them according to space (SSG, LSG) (Casamichana and Castellano, 2010). However, in this study we wanted to include a classification of tasks where, in addition to taking space into account, tactical intentions were considered, as planned in the daily practice of professional soccer. It is considered that most of the specific tasks that can be included in an annual planning could be included within this categorization.

The individual responses were recorded using GPS devices and quantified according to the playing position. Several investigations have described load variables generated in SSG drills (Asian-Clemente et al., 2021; Gonçalves et al., 2016; Köklü et al., 2020; Sangnier et al., 2019), and there are also comparisons between this type of drills and friendly matches (Casamichana et al., 2012). However, the conditional loads of professional soccer players in all the drills that can be used by the coach for two full seasons have not been assessed.

The most important findings of this study indicate that the level of the workload was significantly different according to the soccer-specific drills studied and that the total distance covered, high-speed running and the total number of high accelerations and decelerations were significantly higher in competition than in drills used for training sessions (*p* < .001).

In soccer, one of the main objectives of teams is to reduce own uncertainty and increase, at the same time, the uncertainty of the rival team, generating a constant challenge for players who must develop the capacity of adapting through appropriate technical and tactical abilities (Serra-Olivares et al., 2016). Consequently, coaches must understand that specific training has become their main resource to maximize athletes’ performance (Bradley and Ade, 2018; Gonçalves et al., 2016; Morgans et al., 2018; Owen et al., 2017). In the present study, all soccer-specific drills developed throughout two seasons with professional soccer teams were recorded. These drills were grouped according to their level of technical-tactical specificity (SPG, BPG, SSG and LSG) because they were performed more frequently during training sessions. This finding highlights the importance of training tactical behaviours to achieve the competition objectives (Ade et al., 2016).

This sport requires players to face a complex environment filled with uncertainty and quickly changing, where players must collect information about the contextual and situational elements (ball, teammates, opponents, playing position, moment of the game, etc.). Under these circumstances, players must make the correct decision, often under the pressure produced by spatial-temporal perceptions, respecting tactical objectives and assuming the physical and technical limitations of the action (Serra-Olivares et al., 2016; Vestberg et al., 2012). Based on learning theories, training through specific drills presents opportunities for players to learn to anticipate, make decisions and perform technical skills in a dynamic and highly variable manner, imitating the competitive situation and favouring discovery and learning by trial and error (Williams et al., 2012). In accordance with the principles of neuroplasticity, knowledge structures created as a result of tactical experience, guarantee more effective processing of contextual information (Lex et al., 2015; Williams et al., 2012), thus increasing success in anticipation in competitive situations (Lex et al., 2015; Serra-Olivares et al., 2016; Williams et al., 2012). The relevance and interest of specific drills in modern soccer training can be attributed to the fact that they offer a multifunctional training benefit by influencing simultaneously players’ physical, technical and tactical abilities (Dellal et al., 2012).

The physical, technical and tactical demands of specific drills should be fully understood by coaches, and, at the same time, require care when administering them within a training structure as rule changes may influence the intensity of exercises (Dellal et al., 2012; Hill-Haas et al., 2010). Taking as reference the values of official matches, LSG appear to be a useful mean to understand the players’ performance in competitive environments despite being a relatively unpopular topic in the literature. There is a lot of variability in LSG, however, coaches can adopt different rules to improve the specific skills of soccer players (Dellal et al., 2012).

Soccer players covered greater distances in soccer drills in large spaces (LSG, BPG and PG), as other studies have shown (Asian-Clemente et al., 2021; Casamichana and Castellano, 2010; Casamichana et al., 2012), and these exercises were the ones that most closely resembled the Match Game loads. Literature has shown that SSG do not allow the soccer player to reach high speeds due to space. However, Asian-Clemente et al. (2021) recently indicated the possibility of using SSG with area change to be able to achieve high intensities with the participation of few players in a reduced space. Although the statistical analysis of this study did not show significant differences between the different player positions in soccer-specific drills, the RHSR recorded in BPG and LSG showed similar proportions to those published by Bradley and Ade (2018) according to the specific playing position.

In this study, two types of drills with reduced space were differentiated: SPG and SSG. Although there is directionality and positional roles in both categories of drills, the difference between them is the offensive tactical intention of trying to score a goal or keep the ball, or defensive intention of trying to recover the ball with goals (SSG) or without goals (SPG), as found in another study (Gaudino et al., 2014). SPG showed the lowest load levels according to the physical variables recorded. However, SSG are considered a good strategy to develop the physical qualities of soccer players (Casamichana and Castellano, 2010; Hill-Haas et al., 2010; Köklü et al., 2020; Sangnier et al., 2019). The relevance and interest of SSG in modern soccer training can be attributed to the fact that they offer a multifunctional training benefit by simultaneously influencing the physical, technical and tactical abilities of players (Dellal et al., 2008). Modifying the dimensions of the activity space, the number of players and the rules of the game are the variables that should be considered to manipulate the player's response in favour of the training process (Dellal et al., 2012; Hill-Haas et al., 2010).

In contrast to the results presented by Casamichana et al. (2012), in our study the variable RTDC in SSG did not show significance when comparing this variable with competition matches. However, the analysis showed that the neuromuscular demand in SSG was high, with greater RAc:Dc values in relation to the rest of the drills. In addition, the maximum speed reached in SSG was similar to that recorded in other studies (Asian-Clemente et al., 2021; Casamichana et al., 2012). The number of accelerations and decelerations per minute is an indicator of the ability of players to change speed during the match (Gaudino et al., 2014). Nevado-Garrosa et al. (2021) demonstrated that SSG were an effective training tool to improve variables related to repeated efforts and the ability to maintain acceleration and deceleration over time. However, the space designated to develop these drills limits the ability to reach distances at maximum speeds, and the need arises to increase the number of meters covered per player or use variations to increase the distance (Asian-Clemente et al., 2021; Clemente et al., 2014).

PG and TC had less specific cognitive demand. However, they exhibited higher RHSR values than in SPG and differences between particular playing positions were found. PG are exercises in medium or large spaces without goalkeepers. There are no specific positional roles and their main objective is to maintain possession of the ball. The spaces and the mobility of players in these exercises allow the physical-physiological demands to vary with respect to SSG (Clemente et al., 2014). Only around 10% of the total distance covered was performed with high intensity movements, since most of the activities were carried out at low speed (walking and running); nevertheless, high intensity efforts are decisive in competition (Bradley et al., 2009, 2010; Di Salvo et al., 2007, 2009). In agreement with the principles of training and load variability (Malone et al., 2015), these exercises could be complementary at a conditional level at times of the season where the level of technical-tactical specificity was not the priority (Clemente et al., 2014).

The findings of the present study show that the level of technical-tactical specificity of the exercises seems to be related to the physical response of soccer players depending on their playing position. Although there are publications in which the physical performance of soccer players could be verified based on their playing position using a traditional approach (Bradley et al., 2009, 2010; Di Salvo et al., 2007, 2009), Bradley and Ale (2018) showed that, according to the playing position on the field, physical performance was marked by different complex tactical actions required in competition. This could be the reason why there are differences in the load variability between playing positions in drills most similar to a competition match (BPG and LSG), although they were not significant values. Taking as reference the values of competitive matches, a similar distribution of RHSR was found according to the playing position for LSG and BPG (Figure 3). The efforts in LSG and BPG for each specific position were similar to those required in competition (Ade et al., 2016; Bradley and Ade, 2018). However, these stimuli were not observed in any other exercise. According to the results exhibited in competition (Bradley et al., 2009; Bradley and Ade, 2018; Di Salvo et al., 2009; Mohr et al., 2005), findings of this study indicate that BPG and LSG showed that CM had higher TDCR. FB, WM and CF players in LSG had higher RHSR and top speed than in SSG. These drills are characterized by using a number of players and spaces equal or similar to those of competition. The difference between both is in the presence of goals (LSG) or not (BPG). Scientific literature has demonstrated the validity of this type of the task to work on the great behaviours of the game and the physical demands of competition (Casamichana et al., 2012; Clemente et al., 2014; Sangnier et al., 2019). In addition to physical and basic coordination skills, success in ball sports also depends on how information is processed given the complex context that characterizes them (Vestberg et al., 2012). Therefore, regardless of technical responses induced by different types of exercises, coaches should consider physical consequences derived from training the tactical behaviours of players through soccer-specific drills. According to the results of this study, the technical-tactical specificity of the exercise was a variable to be considered to stimulate soccer players according to the competition demands of each playing position. In this sense, in this study, the most specific tasks (LSG and BPG) were the ones that most stimulated players according to their specific playing position.

In conclusion, soccer is a predominantly tactical sport and must be understood from a complex paradigm, where the physical capacity of soccer players is conditioned by tactical solutions which are processed to respond, often in moments under pressure, to situations that change quickly depending on contextual and situational variables. The findings of the present study showed that the training load could be manipulated by selecting specific exercises that are used, this being the main strategy of coaches to maximize performance in competition according to the characteristics of their players. In addition to the playing area and the number of soccer players participating in the drill, the technical-tactical specificity of the exercise was one variable to be considered in order to stimulate players in a very similar way to what is required of each playing position. The methodology adopted in this study is considered the first step in predicting the desirable load in a soccer training session. Different specific exercises require different workloads, something that has to be taken into account when preparing training sessions. The results of this study can be used to provide knowledge about the type of a training task and its duration in order to achieve the desired stimulus.

Despite the fact that most of the studies published in the literature on this topic only recorded the data of one team, in this study all the tasks used over two full seasons in professional soccer were analysed. A detailed analysis of these drills is pivotal in contemporary soccer as it enables an in-depth understanding of the workload imposed on each player because it has practical implications for the training prescription (Gaudino et al., 2014). Scientists can easily get confused and disorient coaches if they use extensive databases ineffectively and this could lead to the rejection of this proposal. However, the fusion of physical and tactical actions in these databases should increase the interest of coaches rather than overwhelm them (Bradley and Ade, 2018).

## Limitations

This study has several limitations: (1) the number of players that made up the sample was rather small; (2) the study was carried out in a specific professional context; (3) the team studied had specific characteristics; (4) the model and game system was similar throughout the two seasons of the study.

## Practical implications

The present study provides useful information on the physical load of different soccer-specific drills carried out in a professional soccer team during training sessions.

This information can serve as a reference to design training sessions and develop the training plan considering the demands of competition.

On the other hand, given the number of drills analysed, this information could be very relevant for coaches who do not have the possibility to control the external training load and determine what type of drills may be most appropriate depending on the objective set, time of the season and team characteristics.
